# The relationship between neck circumference and gestational diabetes mellitus in Iranian women

**DOI:** 10.1186/s40842-021-00136-5

**Published:** 2021-12-14

**Authors:** Tahoora Sedighi Barforoush, Reza Ghadimi, Zaynab Pahlevan, Niloufar Ahmadi, Mouloud Agajani Delavar

**Affiliations:** 1 Student Committee Research, Babol University of Medical Sciences, Babol, Iran; 2 Department of Community Medicine, School of Medicine, Social Determinants of Health, Research Center, Research Institute for Health, Babol University of Medical Sciences, Babol, Iran; 3 Department of Obstetrics & Gynecology, Infertility and Reproductive Health Research Center, Research Institute for Health, Babol University of Medical Sciences, Babol, Iran; 4 Infertility and Reproductive Health Research Center, Research Institute for Health, Babol University of Medical Sciences, Babol-Amol oldhighway, after Mohammadhasan Khan Bridge, Po. Box: 47135-547, Babol, Mazandaran Iran

**Keywords:** Gestational diabetes mellitus, Pregnancy, Neck circumference

## Abstract

**Background:**

The aim of the present study was to assess the relationship between neck circumference and gestational diabetes.

**Methods:**

This prospective study was conducted on 372 Iranian pregnant women. The criteria set by the American Diabetes Association through 2 h was used to classify subjects with regard to their gestational diabetes. At the 14–16th weeks of pregnancy, the neck circumference was measured. The maternal and fetal outcomes were measured as well.

**Results:**

The adjusted logistic regression revealed that neck circumference was a predictor for gestational diabetes mellitus (OR = 1.20; 95% CI = 1.06, 1.34; *P* = 0.002). The ROC analysis depicted that the cut-off for neck circumference in indicating gestational diabetes was 34.3 cm, with the sensitivity of 53% and the specificity of 66%.

**Conclusion:**

The findings of the present study revealed that the neck circumference of ≥34.3 cm can be deemed as a predictor of gestational diabetes in the case of Iranian pregnant women.

## Background

Gestational diabetes mellitus, first recognized at 24-28th weeks of gestation, is a form of oral glucose intolerance, affecting 1 to 14% of pregnancies worldwide [1]. The prevalence of gestational diabetes mellitus among Iranian women has been reported differently by various scholars (from 3.1 to 18.6%) [2], its prevalence is currently growing worldwide [3, 4]. It is worth noting that women who are treated with insulin in pregnancy are more likely to face the risk of developing diabetes [5–7] as well as cardiovascular problems after pregnancy [8–10]. Nonetheless, the timely prediction of gestational diabetes and the start of an early effective intervention in the first or second trimester may mitigate the risk of gestational diabetes and yield good results for both the fetus and the mother [11, 12]. It is generally assumed that gestational diabetes could be associated with adverse fetal, infantile, and maternal outcomes such as the sustained impairment of glucose tolerance, preeclampsia, macrosomia, neonatal hypoglycemia, neonatal death caused by respiratory distress, and shoulder dystocia [13–17]. Numerous studies recommend that meticulous strategies including glucose tolerance test [18], ultrasonographic adipose tissue thickness [19], and HbA1c screening [19, 20] should be developed at the first trimester of pregnancy for the prediction of gestational diabetes mellitus, which can help reduce the risk of gestational diabetes in high risk women. However, there is still no consensus on appropriate screening strategies for gestational diabetes mellitus [21], and many of the tools used in the Western world are inaccessible to women in the developing world. Thus, it could be regarded as a potential risk factor for the development of metabolic syndrome [22], cardiovascular diseases [23], maternal obesity, and maternal type 2 diabetes after pregnancy [24]. In addition, previous studies have demonstrated the presence of an element of metabolic syndrome among pregnant women in gestational diabetes mellitus, suggesting that the risk factors for diabetes mellitus and metabolic syndrome are the same [25–27]. The most prevalent risk factors for metabolic syndrome are: waist-to-hip ratio, hip circumference, and waist circumference [28]. Neck circumference was also reported to be a marker of fat distribution over the trunk and also has a clear association with waist-to-hip ratio, waist circumference, body mass index, and glycemic status among non-pregnant women [29, 30]. Neck circumference was also reported to be clearly associated with increased plasma-free, fatty acid levels [31]. It is thought that neck circumference could be a better marker than waist circumference or any other markers for the determination of metabolic syndrome and its key features. Its measurement is also both convenient and reproducible [21, 26, 32].

According to Hoebel et al., neck circumference can be a helpful marker for not only metabolic syndrome but also its risk factors including central obesity, insulin resistance, triglycerides, and fasting blood sugar [27]. In the light of these facts, it can be hypothesized that the risk of gestational diabetes can increase among pregnant women with higher neck circumference. Also, it can be postulated that the need to identify and provide healthcare for pregnant women in Iran should currently be deemed as a top priority for physicians. Therefore, the aim of this study was to assess the relationship between neck circumference and gestational diabetes mellitus during the prenatal care visits of pregnant women.

## Methods

The study was approved by the ethics committee of Babol University of Medical Science (Ethic ID: IR.MUBABOL.HRI.REC.1398.022). The minimum sample size for this study was calculated to be around 411, which was based on the estimated prevalence of 18.6% in Tehran, Iran [33], with a standard score of 95%, the power of 80%, the margin error of 4, and 15% of drop-out rate.

A total of 411 singleton pregnant women, who were between 18 and 35 years of age, were recruited for this study at prenatal clinics affiliated with Babol University of Medical Sciences. All the recruited women were at 14–16th weeks of pregnancy. This prospective study was in progress over the period between March 2019 and February 2020. The women with a history of pre-gestational diabetes (*n* = 11), dyslipidemia (*n* = 2), chronic hypertension (*n* = 4), thyroid disease (*n* = 6) and other endocrine diseases (*n* = 2), fetal malformations in nuchal translucency (NT) (*n* = 2), and hyperglycemic drugs (corticosteroids and thyroid hormones) (*n* = 1) were excluded from the study. Twenty-four women were initially excluded from the study, which was based on the results of the routine prenatal blood tests at the first trimester of pregnancy as well as the information obtained from their medical records. Therefore, the remaining 387 eligible women signed the written informed consent forms. The data for all pregnant women were collected at three time-points: 14–16th gestational weeks, 24-28th weeks, and at baby delivery.

During the 14–16th weeks of pregnancy, the characteristics of the participants such as age, gravidity, neck circumference, and the family history of diabetes were obtained. The women were requested to report the pre-pregnancy weight (self-report). If the woman did not remember the weight before her pregnancy, the weight of the first visit (first trimester of pregnancy) was recorded. The gestational age of the participants was defined according to the last menstrual period and established by an early ultrasound of the pregnancy. The height was measured with a tape measure without shoes. The body mass index was measured by the subsequent formula: weight (kg)/ height squared (m^2^). The neck circumference was determined through a tape from the level just below the larynx (accuracy 1 cm) with subjects in standing position, with a straight head and shoulders [34].

At 24-28th weeks of pregnancy, the blood pressure (BP) was measured with calibrated mercury sphygmomanometers with appropriate size cuffs after the women had rested for 15 min. The Korotkoff phase 1 (first sound) and Korotkoff phase 5 (fifth phase) of blood pressure were defined as systolic blood pressure and diastolic blood pressure, respectively [35]. Pregnancy induced hypertension (PIH) was defined as blood pressure greater than or equal to 140/90 mmHg, with or without proteinuria during pregnancy [36]. In addition, a two-hour, 75-g oral glucose tolerance test (OGTT) was performed after a ten-hour fasting in sitting position. All blood samples were analyzed at laboratories affiliated with Babol University of Medical Sciences. If there were any of the following glucose cut-off levels: fasting ≥92 mg/dl or one-hour ≥180 mg/dl or and two-hour ≥153 mg/dl, the women were diagnosed with gestational diabetes [5]. Out of 387 women who were followed until child birth, 15 women with diagnosed pregnancy-induced hypertension and thyroid disease were excluded from the study for the accurate assessment of the relationship between neck circumference and gestational diabetes.

At delivery, the maternal weight at the end of the pregnancy, the type of birth, the weight of the newborn, the fetal respiratory syndrome, and the admission to the neonatal intensive-care unit (NICU) were obtained.

### Statistical analysis

All the analyses were performed by SPSS software version 20.0 (SPSS Inc., Chicago, IL, USA). The Kolmogorov-Smirnoff test was used as a test for evaluating the normality of the dataset. The demographic and anthropometric characteristics, blood pressure and blood glucose (gestational diabetes mellitus) were compared for the two groups using independent t-test and chi-square test. The correlation between neck circumference and the risk factors of gestational diabetes mellitus was assessed by Pearson coefficient test. Age adjusted binary logistic regression analysis was used for present odds ratio (OR) and confidence interval (95% CI). Also, ROC analysis was used to evaluate the predictability of gestational diabetes. The area under the curve was calculated by SPSS software, with due sensitivity and specificity, we strove to obtain the best neck circumference cut-off points. The significance level for all tests was < 0.05.

## Results

The analysis included 372 participants with a mean age of 28.1 ± 4.4 years. The participants had a mean height, weight, and body mass index of 162.0 ± 5.6 cm, 69.9 ± 9.9 kg, 26.6 ± 3.4 kg/m^2^, respectively. The mean neck circumference was calculated to be 34.3 ± 2.3 cm at 14–16th weeks of pregnancy. According to the criteria set by the American Diabetes Association through 2 h [37], the gestational diabetes mellitus was diagnosed in 74 of the participants; consequently, the participants were classified into two groups: the women with gestational diabetes (*n* = 74), as a case group, and those with normal pregnancies, without gestational diabetes (*n* = 298), as a control group. Among women in the case group, 45.9% (*n* = 34) of them were treated with insulin during pregnancy, and only one received metformin. The remaining women in the case group underwent diet therapy during pregnancy. It is worth noting that high gravidity, neck circumferences, pre-pregnancy body mass indices, and the family history of type 2 diabetes were significantly higher in women with gestational diabetes compared with those of the normal group. Also, women with gestational diabetes experienced a significant increase in fasting blood sugar, 1-h glucose, and 2-hrour glucose compared with those in the normal group (Table 1).

**Table 1:** Characteristics of the participants by the gestational diabetes mellitus

Variables	Gestational Diabetes Mellitus *n* = 74 Mean ± SD	Normal *n* = 298 Mean ± SD	*P* value
Age (years)	161.7 ± 0.5	162.1 ± 5.7	0.578
**Gravidity n (%)**			< 0.001
One	20 (27.0)	152 (51.0)	
≥ 2	54 (73.0)	146 (49.0)	
Family history of type 2 diabetes n (%)	26 (35.1)	24 (8.1)	< 0.001
Pre-pregnancy weight **(**Kg)	71.6 ± 10.6	69.5 9.7	0.092
Height (cm)	161.7 ± 5.0	162.1 ± 5.7	0.578
Pre-pregnancy BMI^a^	27.4 ± 4.0	26.4 ± 3.2	0.045
Neck Circumference	35.1 ± 2.7	34.1 ± 2.1	0.005
Systolic BP^b^ (mmHg)	110.5 ± 10.0	108.7 (10.5)	0.180
Diastolic BP^b^ (mmHg)	70.2 (8.6)	68.7 (9.2)	0.643
Weight gain during pregnancy (Kg)	11.0 ± 4.3	11.4 ± 4.3	0.506
FBS ^c^(mg/dl)	106.8 ± 23.5	81.5 ± 5.8	< 0.001
1-h glucose (mg/dl)	124.5 ± 43.6)	102.4 ± 17.3	< 0.001
2-hrour glucose (mg/dl)	128.9 ± 36.9	97.5 ± 15.2	< 0.001

^a^*BMI* Body mass index, ^b^*BP* Blood pressure, ^c^*FBS* Fasting blood glucose

The results of Pearson correlation depicted that neck circumference was significantly correlated with age, pregnancy weight gain, maternal weight pre-pregnancy, body mass index, fasting blood sugar, and OGTT two-hour Glucose (Table 2).

**Table 2:** Pearson’s correlation between neck circumference and risk factors of gestational diabetes mellitus

Variables	r	*P* value
Age (years)	0.18	≤0.001
Pre-pregnancy weight (Kg)	0.45	≤0.001
BMI^a^	0.46	≤0.001
Weight gain during pregnancy (Kg)	0.11	0.031
FBS (mg/dl) ^b^	0.232	≤0.001
1-h glucose (mg/dl)	0.172	0.001
2-hrour glucose (mg/dl)	0.034	0.518

^a^*BMI* Body mass index, ^b^*FBS* Fasting blood glucose

Table 3 illustrates the estimated adjusted odds ratio (with 95% CI) of gestational diabetes and the independent variables. Accordingly, neck circumference (OR = 1.20; 95% CI = 1.06, 1.34), BMI (OR = 1.08; 95% CI = 1.01, 1.16), 2-hrour glucose (OR = 1.05; 95% CI = 1.04, 1.06), 1-h glucose (OR = 1.03; 95% CI = 1.02, 1.04), and FBS (OR = 1.40; 95% CI = 1.28, 1.53) were the independent variables for gestational diabetes mellitus.

**Table 3:** Adjusted^a^ odds ratio for gestational diabetes mellitus and dichotomous variables (*n* = 372)

Variables	Odds ratio	95% Confidence interval	*P* value
Pre-pregnancy weight (Kg)	1.02	1.00–1.05	0.103
BMI^b^	1.08	1.01–1.16	0.031
Weight gain during pregnancy (Kg)	0.97	0.91–1.03	0.266
FBS (mg/dl)^c^	1.40	1.28–1.53	< 0.001
1-h glucose (mg/dl)	1.03	1.02–1.04	< 0.001
2-hrour glucose (mg/dl)	1.05	1.04–1.06	< 0.001
Neck circumference	1.20	1.06–1.34	0.002

^a^Adjusted for age, ^b^*BMI* Body mass index, ^c^*FBS* Fasting blood glucose

**Fig. 1:**
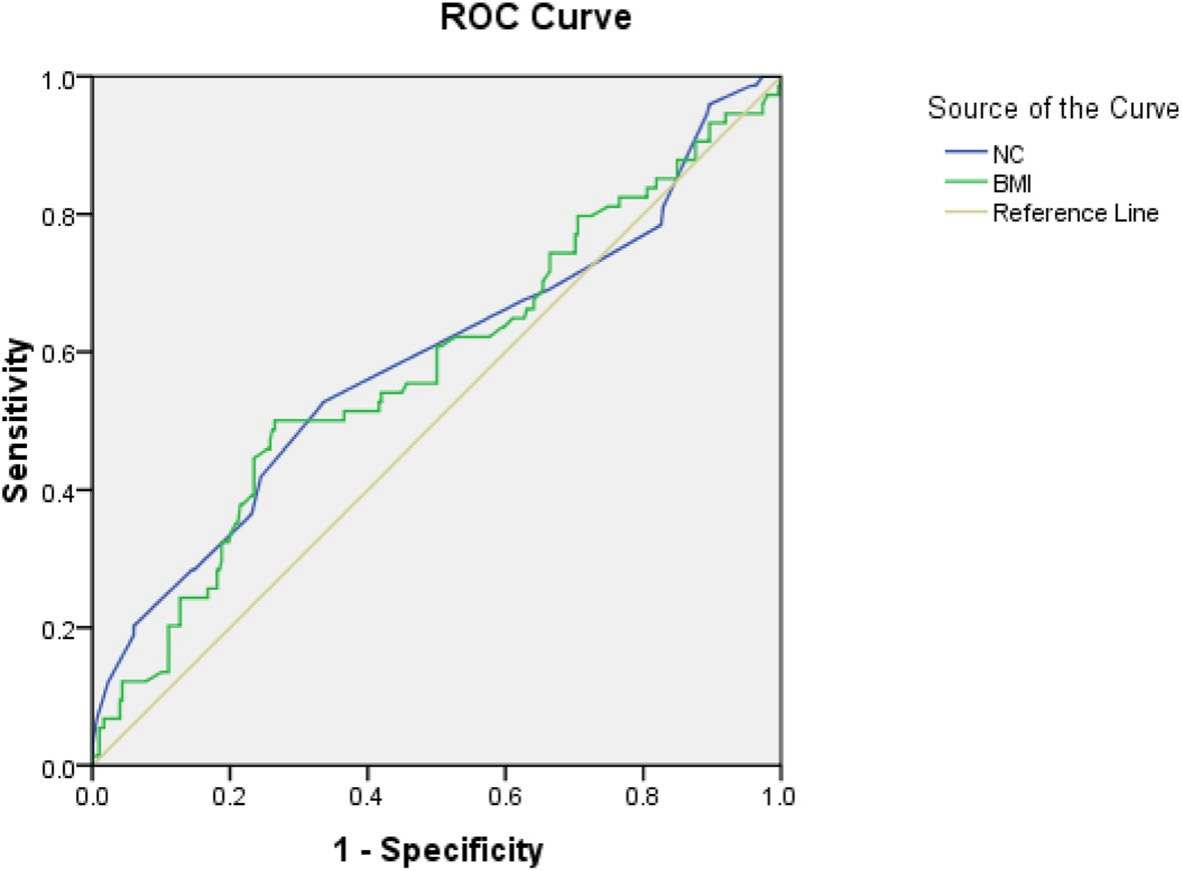
Receiver operating characteristic (ROC) curve of neck circumference (*n* = 372)

The ROC analysis demonstrated that the optimal cut-off value for neck circumference and body mass index before pregnancy in gestational diabetes mellitus was 34.3 cm with the sensitivity of 53% and the specificity of 66% and 26.5 kg/m^2^ with the sensitivity of 54% and the specificity of 58%, respectively. Moreover, the areas under the curve of neck circumference and body mass index before pregnancy were 0.59 (95% CI 0.51–0.67) and 0.58 (95% CI 0.50–0.65), respectively (Fig. 1).

There was a significant association found between fetal distress syndrome and gestational diabetes mellitus. The risk of fetal distress syndrome and admission to NICU for women with gestational diabetes was higher than those of the normal group (OR = 4.29; 95% CI = 1.34, 13.73) and (OR = 3.04; 95% CI = 1.26, 7.40), respectively. There was no statistically significant association between the two groups in terms of delivery type and newborn weight and neck circumference (Table 4).

**Table 4:** Maternal and neonatal outcome of participants by gestational diabetes (*n* = 372)

Variables	Gestational Diabetes Mellitus *n* = 74 N (%)	Normal *n* = 298 N (%)	Odds ratio	95% CI	*P* value
**Type of delivery**					0.459
Cesarean	45 (60.8)	167 (56.0)	1.22	0.72–2.05	
Vaginal	29 (39.2)	131 (44.0)	1.00		
**Fetal distress syndrome**					0.014
**Yes**	6 (8.1)	6 (2.0)	4.29	1.34–13.73	
**No**	68 (91.0)	292 (98.0)	1.00		
**Administration NICU**^a^					0.015
**Yes**	9 (12.2)	13 (4.4)	3.04	1.26–7.40	
**No**	65 (87.8)	285 (95.6)	1.00		
**Newborn weight (gr)**					0.273
< 2500 or > 4000	6 (8.1)	38 (12.8)	0.604	0.25–1.49	
2500–4000	68 (91.9)	260 (87.2)	1.00		

^a^*NICU* Neonatal intensive-care unit

Table 5 presented the association between the delivery type and neck circumference in pregnant women. Accordingly, the risk cesarean and abnormal FBS at 24-28th weeks in women with the neck circumference of ≥34.3 cm was higher compared with those who had the neck circumference of < 34.3 cm (OR = 1.59; 95% CI = 1.03, 2.45) and (OR = 2.91; 95% CI = 1.66, 5.10), respectively.

**Table 5:** Maternal and neonatal outcome and OGTT value of participants by neck circumference (*n* = 372)

Variables	NC < 34.3 cm *n* = 233 N (%)	NC ≥ 34.3 cm *n* = 139 N (%)	Odds ratio	95% Confidence interval	*P* value
**Type of delivery**					0.035
Cesarean	123 (52.8)	89 (64.0)	1.59	1.03–2.45	
Vaginal	110 (47.2)	50 (36.0)	1.00		
**Fetal distress syndrome**					0.363
**Yes**	6 (2.6)	6 (4.3)	1.71	0.54–5.40	
**No**	227 (97.4)	133 (95.7)	1.00		
**Administration NICU**^a^					0.211
**Yes**	11 (4.7)	11 (7.9)	1.73	0.73–4.11	
**No**	222 (95.3)	128 (92.1)	1.00		
**Newborn weight (gr)**					0.633
< 2500 or > 4000	29 (12.4)	15 (10.8)	0.85	0.44–1.65	
2500–4000	204 (87.6)	124 (89.2)	1.00		
**FBS (mg/dl)**^b^					< 0.001
Abnormal	25 (10.7)	36 (25.9)	2.91	1.66–5.10	
Normal	208 (89.3)	103 (74.1)	1.00		
**1-h glucose (mg/dl)**					0.151
Abnormal	10 (4.3)	2 (1.4	0.33	0.07–1.51	
Normal	223 (95.7)	137 (98.6)	1.00		
**2-hrour glucose (mg/dl)**					0.567
Abnormal	17 (7.3)	8 (5.8)	0.78	0.33–1.85	
Normal	216 (92.7)	131 (94.2)	1.00		

^a^*NICU* Neonatal intensive-care unit, ^b^*FBS* Fasting blood glucose

## Discussion

Drawing on the results of the present study, we can claim that thicker neck circumferences could be associated with a higher risk of gestational diabetes mellitus in pregnant women (18–35 years of age) with a reported probability of pregnancy risk of 1.20 in Iran. Consistent with our finding, there is a study by Li et al. (2018), conducted on 371 Chinese pregnant women in China (97 diabetic and 274 non-diabetics). They concluded that neck circumference was an independent predictor of gestational diabetes mellitus. The probability of pregnancy risk was also reported at 1.29, using binary logistic regression [38]. In another study, He et al. (2017) conducted a nested case-control study on 255 pregnant women (41 with diabetes and 214 without diabetes), aged 18–35, in China. These authors found that neck circumference predicted gestational diabetes mellitus at the 16th week of gestation as an independent variable. Therefore, the probability risk for gestational diabetes mellitus was reported to be 1.80, which was slightly higher than that of our study [39].

We also found that neck circumference was positively correlated with all the risk factors of gestational diabetes except for the 2-h glucose, which was in agreement with the results of previous studies [39, 40]. In recent years, several studies have been conducted on pregnant women in China [38, 39] and Pakistani [40]. These research studies strove not only to assess the correlation between neck circumference and gestational diabetes but also to estimate the optimal cut-off value of neck circumference. It appears that the detection of a cut-off value for neck circumference, which can help identify gestational diabetes, is a big step forward for the care of pregnant women worldwide. In contrast to the results of two previous studies on Chinese women [38, 39] and a study on women from Pakistan [40], we found that the calculated cut-point of neck circumference for the prediction of gestational diabetes mellitus was lower (34.3 in Iranian women vs. 33.8 cm in Chinese women, 35.2 cm in Han Chinese women and 35.7 cm in Pakistan women), with varying sensitivity and specificity values. A possible explanation for this discrepancy may be the sample size, the design of the study, and the ethnicity of the participants.

### Limitations

There were three limitations in our study. To begin with, all the participants were selected only from the prenatal clinics affiliated with Babol University of Medical Sciences. Future studies should be conducted in various centers and on larger samples so that they can provide stronger evidence for potential associations. Secondly, although most pregnant women had medical documents in clinics, the use of self-reported pre-pregnancy weight to calculate the pre-pregnancy body mass index might have led to an inevitable data quality problem. This is perhaps an unavoidable issue in many other studies, as it is hard to measure the weight of women before the onset of the study. Third, we did not evaluate the prediction for the development of type 2 diabetes and/or metabolic syndrome in women with gestational diabetes. In addition, information on women with a history of gestational diabetes mellitus was not available because women with a history of gestational diabetes were excluded from the study. Future studies with longer follow-up periods need to be conducted to provide stronger evidence for this potential association.

## Conclusion

Despite all these limitations, our study used a prospective design to assess the relationship between neck circumference and gestational diabetes mellitus. According to the results, neck circumference can be recommended as a predictor of gestational diabetes mellitus, although the test has low sensitivity (sensitivity reported from 40.8 to 64.8%) and a partially high specificity (specificity reported from 60.8 to 71.8%), with the effect size of 0.14 and 80% actual power. In addition, using neck circumference for screening gestational diabetes mellitus has shown a low positive predictive value and high negative predictive value, so these cannot affect the applicability of the findings. To illustrate this, it is worth mentioning that the sensitivity and specificity of neck circumference for predicting gestational diabetes mellitus in our study were similar to those of pre-pregnancy body mass index. Additionally, we should bear in mind that body mass index measure could have its own limitations. This study illustrated that Iranian pregnant women with a neck circumference of ≥34.3 cm were more likely to develop gestational diabetes. Thus, we can conclude that the result of this study may be used as a basis for predicting gestational diabetes in Iran.

## Data Availability

Not applicable.

## Abbreviations

BP: Blood pressure
NT: Nuchal translucency
OGTT: Oral glucose tolerance test
NICU: Neonatal intensive-care unit
